# Bayesian hierarchical model predicts biopharmaceutical stability indicators and shelf life with application to multivalent human papillomavirus vaccine

**DOI:** 10.1038/s41598-025-99458-y

**Published:** 2025-05-19

**Authors:** Federico Ferrari, Jordan Berger, Linda Lemieux, Crina Paduraru, Michael Dillon, Andy Liaw, Ralf Carrillo, Sally Wong, Hossein Salami, Paolo Avalle, Edward Sherer, Douglas Richardson, Daniel Skomski

**Affiliations:** 1 Merck & Co., Inc., Rahway, NJ USA; 2 Merck & Co., Inc., West Point, PA USA; 3 Merck & Co., Inc., Cambridge, MA USA; 4 MSD Werthenstein BioPharma GmbH, Lucerne, Switzerland

**Keywords:** Biological techniques, Biotechnology, Computational biology and bioinformatics

## Abstract

Predictive stability is demonstrated as a powerful method for assessing the shelf-life of biopharmaceutical products, such as therapeutic proteins and vaccines. A Bayesian hierarchical multi-level stability model is illustrated for the Human Papillomavirus (HPV) 9-valent recombinant sub-unit vaccine GARDASIL®9. Ensuring speedy manufacturing and ample supply to satisfy the need of patients globally is pivotal, particularly for expanding vaccine access to underserved populations. Product heat stability and cold-chain supply play a major role in deployment of vaccines particularly to lower income countries, while lengthy real-time stability and shelf-life supporting studies are resource-intensive and time-consuming. Hence, an accelerated model-informed stability approach is devised. The product in this case study contains 9 molecular types (antigens) which each target different viral genotypes of the same class of the virus. The molecular types are mixed together as a co-formulation within a container (vial or syringe). The stability behavior of the platform vaccine was well-characterized experimentally and a single stability-limiting attribute was identified (potency). A Bayesian hierarchical stability model was developed utilizing long-term drug product storage data through shelf life at 5 °C as well as shorter-term accelerated stability data at 25 °C and 37 °C for 30 product batches. The model was able to comprehensively assess the stability of all molecular types in the vaccine as well as covariates like container type within a singular unified model framework. Moreover, method superiority was demonstrated for this application over multiple approaches such as linear and mixed effects models. This work elucidates that biopharmaceutical product platform knowledge from previous lots of a biopharmaceutical product in conjunction with batch-specific data from early stability timepoints can be employed to support long-term assessment for shelf-life of the stability and shelf-life indicating attribute(s). These findings, applied to two types vaccines including a multivalent vaccine, hold utility towards enabling accelerated patient access of future complex vaccines and biotherapeutic modalities. The results provide a novel framework for estimating a model for stability data in the context of evolving regulatory guidance.

**Supplementary Information:**

The online version contains supplementary material available at 10.1038/s41598-025-99458-y.

## Introduction

### Motivation

HPV is a common sexually transmitted infection that can be implicated in the formation of various cancer types, including cervical, vulvar, vaginal, anal, penile, and oropharyngeal cancers, as well as genital warts^1^ GARDASIL®9 has significantly aided prevention of HPV-related diseases. Clinical trials have demonstrated the efficacy of GARDASIL®9 in preventing infections and diseases,^1^ leading to its contribution towards reducing HPV-related morbidity and mortality. Demand for HPV vaccination is expected to continue to climb particularly involving a need to increase vaccine coverage and accessibility in less developed regions^2^.

The World Health Organization (WHO) has articulated an urgent need around technological innovations for vaccines, including modelling, to address storage condition management to increase patient access, improve vaccine performance, lower cost, and increase flexibility^3^ Heat stability, cold-chain supply management, and resultant avoidable wastage are major issues in deployment of vaccines to lower and middle income countries^3,4^ An area of particular importance is to address information uncertainty in understanding temperature limitations of vaccines^5^ Predictive stability and shelf-life prediction for new pharmaceutical product batches are believed to play a role in the goal to accelerate patient access of current and future vaccines^6^.

Historically, Q1/Q5C have provided limited guidance for how much extrapolation is appropriate for determining shelf life while focusing on simple regression analysis in setting shelf-life. The extrapolation techniques described are limited in scope, and by definition are not able to incorporate multi-factor analysis beyond a given trend at a given set of conditions. To address these deficiencies, proposals towards incorporation of enhanced stability modelling^7^ have been endorsed by the International Council for Harmonisation of Technical Requirements for Pharmaceuticals for Human Use (ICH)^8^ for inclusion in the industry guidelines. The ICH guidelines Q1A/Q5C are undergoing revision to include an annex for stability modelling and model-informed shelf-life setting inclusive of Bayesian statistics and other complex models that take into account multiple factors of stability-indicating information. Prediction intervals described in this work can adjust for multiple variables and scenarios and affirm the continued validity of trends. Hence the outlined approach is not only aligned with the evolving regulatory landscape, but also specifically highlights the gap that the guideline update will reflect.

Real-time pharmaceutical stability studies in support of shelf-life and regulatory submissions are time-limiting and resource-intensive in biopharmaceutical research and development. Methods to predict long-term stability in order to accelerate time to deployment to patients of novel biopharmaceutical medicines and vaccines are of great interest to industrial, government, and patient-centric stakeholders. The aspirational state of predictive stability methods is to enable rapid patient access to new medicines and vaccines by accurately predicting and establishing drug product expiry/shelf-life faster and with fewer resources. The aim is to demonstrate that this future strategy can be accomplished by leveraging less than the historically acceptable amount of stability experimental data across modalities, while maintaining the same confidence of a product meeting critical quality attributes through shelf-life in regulatory filings. It is also of interest to reduce reliance on large formal stability studies to address changes in e.g. product, process, container-closure components, etc. This would serve the goal of faster time to the clinic for patients in need and more rapid access to new cutting-edge therapies.

### Applications of predictive stability for pharmaceutical shelf-life

Stability is critical to product quality and pivotal towards regulatory expectations from health authorities. As stability is essential throughout the entire pharmaceutical development and commercialization landscape, and the understanding thereof evolves during the product lifecycle, predictive stability technologies and methodologies hold enormous potential for saving significant time as well as human and material resources. In the context of greater future adoption and acceptance of such methods, the utilization of these tools could lead to a decrease in the workload associated with experimental testing throughout the various complex stages, encompassing material production, sample preparation, stability testing at multiple time points, data acquisition, analysis and reporting, formal documentation creation, and additional studies conducted throughout the duration of a product’s lifespan. While model validation studies would still be needed, including the expectation around formal real-time data, predictive technologies are beneficial to accelerate development of new medicines, reduce experimental testing, and leveraging model-informed risk management.

Some of the benefits of predictive stability are elucidated: (1) Predict stability profiles across conditions & time points based on a smaller experimental data set (for example, use short-term and/or accelerated stability studies to predict long-term shelf-life) including for in-use period, time out of refrigeration, and vial monitor labels. (2) Leverage historical prior platform knowledge for molecular attributes for similar construct or analogous molecular family. (3) Inform risk posture around product liabilities (low, medium, and high risks) for proactive identification and improved management of risks. (4) Build fundamental understanding of the physicochemical degradation pathways leading towards instability versus stability of new molecular entities. (5) Screen and optimize product compositions more quickly by better characterizing and projecting the role of excipients in enhancing or suppressing overall stability while identifying the conditions that produce the greatest overall stability profile. (6) Quantitatively interrogate the relationship of co-factors like process conditions, container-closure systems, etc. onto stability profiles. (7) Better address process and analytical variability and interrogate/deconvolute these factors in relation to stability trends. (8) Improve stability model prediction confidence and statistically rationalize outliers within the range of model variance. (9) Improve predictability on commercial manufacture robustness and the feedback loop to drug discovery. (10) Apply advanced statistical and machine learning algorithms to identify composition and process variables likely to require strict controls in order to achieve consistent stability throughout the product lifecycle.

It is known that compounds with fewer molecular liability risks have greater overall physicochemical stability leading to higher probability of success of completing clinical trials and becoming commercial products,^9^ hence new methods to predict stability or validate intrinsic molecular risks would be of significant benefit. To facilitate risk-based scientific assessment of stability the means to better leverage predictive stability is a topic under extensive discussion^10^ Industry is particularly interested in expanding application of predictive stability approaches to large molecule products like vaccines and biologics as these domains are growing as a proportion of the industry’s overall portfolio of modalities. Towards this goal, institutions would like to expand applications of various predictive and stability modelling approaches^11,12^.

### Existing approaches

Significant progress has been made in predictive stability for small-molecules. Regulatory acceptance has been achieved in the small-molecule domain^10^ for setting initial shelf-life, initial retest period, shelf-life of a formulation variant, justifying storage conditions for drug substance (DS) from a new process, and gauging impact of drug product (DP) process changes on shelf-life. Historically applications in the large-molecule domain have lagged the small-molecule domain, yet synthetically or biologically derived large molecules are assuming an increasing share of the overall biopharmaceutical pipeline. Nevertheless, significant progress in large molecule predictive stability has been made over the past several years. Investigations in predictive stability for large molecules have been focused on experimental approaches,^13–16^ excipient^17^ and adjuvant^18^ driven interactions, and specific mechanisms like aggregation-driven behaviour,^19,20^ as well as improvements to preliminary discovery developability assessments and methodologies^21,22^.

A comprehensive assessment of stability-impacting factors is critical for any predictive model. A multitude factors and their relative importance must be considered. For example, it was shown that small changes in conformational structure and colloidal stability of a given formulation may not necessarily be predictive of long-term instability of high molecular weight species formation (aggregation)^23^ Moreover, stability also needs to be assessed in a holistic context (e.g., beyond change in potency, aggregation, etc.) in relation to other key manufacturing and clinical considerations. For example, it was shown that different combinations of antigen and adjuvants in a vaccine candidate could result in a better stability profile while simultaneously resulting in suboptimal immune responses^24^ Thus modelling techniques are best adopted when they are executed with close integration to experimental methods^25^ and in the context of broader biopharmaceutical manufacturing methodologies^26–29^.

Large-molecule adoption of predictive stability in the biopharmaceutical development context has been proposed for biologics^30^ and vaccines^31^ alongside a universal modelling approach^32^. Because the relationship between storage stability and the performance of vaccine or biologic drug products is commonly empirically evaluated, this field stands to benefit as predictive stability models are better tuned beyond conventional approaches. Particularly in the context of the importance around future pandemic and global health emergency readiness,^6^ stability modelling has benefits to provide shelf-life estimations for vaccines. Typically, such products are stored at cold temperatures, product process/formulation changes are generally more challenging in terms of bridging, and the chemistry of the materials involved and associated degradation pathways are often complex.

### New approach and advantages

**Scheme 1:**
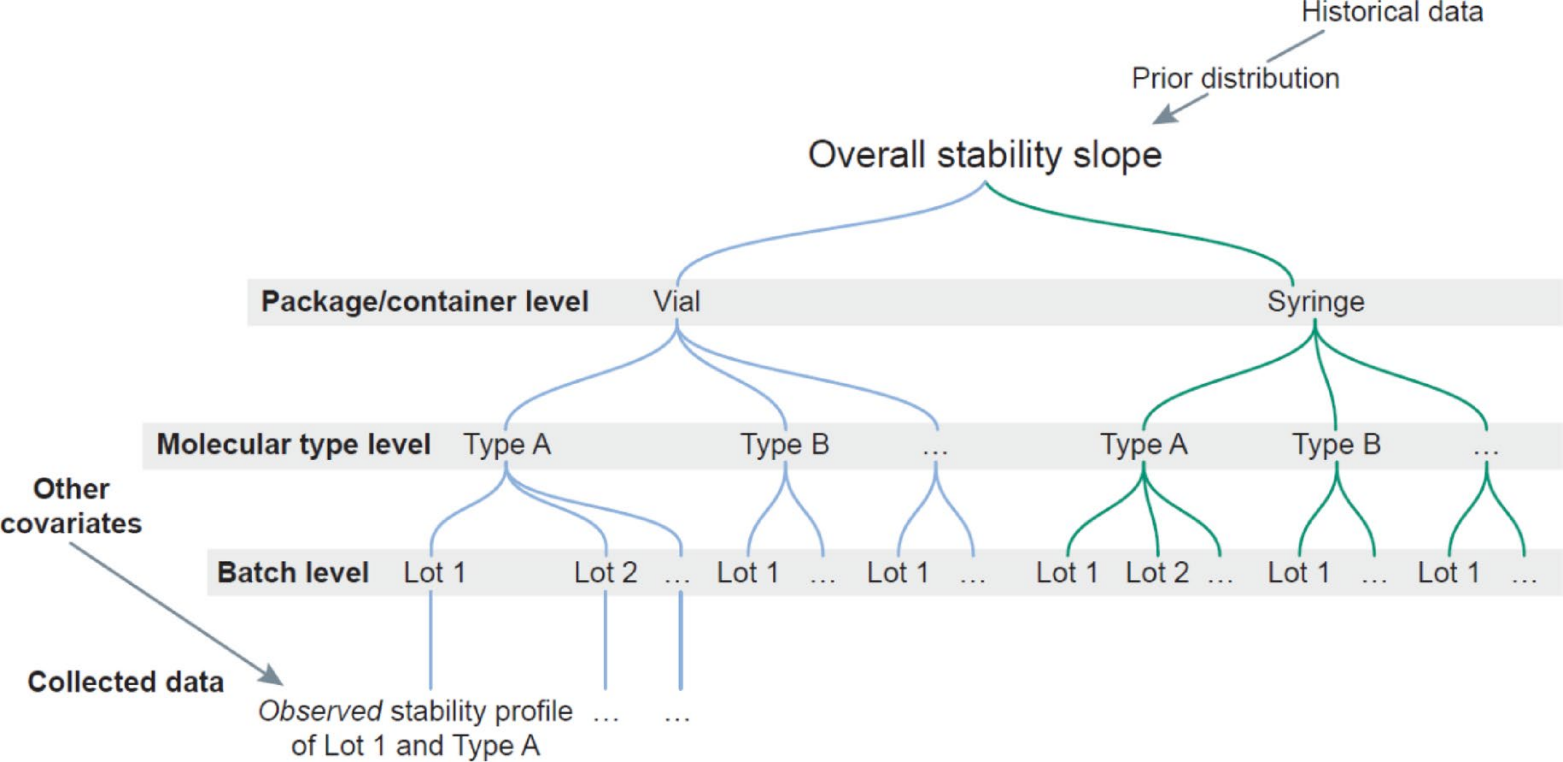
Representation of the Bayesian predictive stability model for the slope parameter. A detailed description of the model is included in the section ‘Model Specification’ in Supplementary Materials.

Given its ability to integrate complex multi-variate datasets,^33^ provide coherent predictions, and generate credible interval estimates to quantify uncertainty, Bayesian statistical models have been successfully employed for stability studies. More specifically, hierarchical Bayesian models can incorporate multiple levels of information, such as different batches, HPV types, or package/container, in a “tree” like structure to estimate parameters of interests and predict outcomes (Scheme 1). For this reason, Bayesian hierarchical models are able to address complex data structures (for example integrating information about different related sub-groups) as well as possessing an intrinsic mechanism for leveraging prior knowledge.

Due to the inherent complexity of large molecule formulations, as well as co-formulation of multiple drug substances in some products and multivalency common in vaccines, universal acceptance and adoption of such predictive tools was rarely adopted in the past but have now become an active consideration with global regulatory bodies amidst an evolving landscape of industry guidelines on stability monitoring and model-based prediction.

Multiple technical, quality, and regulatory barriers had historically existed for implementation of large-molecule predictive stability application for clinical and commercial products. This paper aims to propose and support how to overcome many of these hurdles.

First, for late-stage R&D and commercial line of sight it is essential to provide true quantitative readouts of expected long-term stability and predicted shelf-life in relation to the stability specifications that are set for the drug product on the basis of traditional stability studies and statistical analysis. Advanced Bayesian hierarchical models provide absolute quantitative predictions of stability as well as additional data interpretation that can potentially enhance the control strategy by deriving more accurate predictions from less data, intuitive descriptions of credible intervals, greater insights into covariate relationships, and creating a singular unified model framework to address multiple types of questions. In this paper we present a fully quantitative Hierarchical Bayesian kinetic model capable of predicting stability and subsequent shelf-life in relation to drug product stability specifications set for an HPV platform vaccine product.

Second, it is desirable to better incorporate historical data in models, particularly for well-established “legacy” commercial products or around a molecular “franchise” composed of one drug substance shared and combined across multiple drug product formulations and combinations. For example, historically multiple HPV vaccines have existed with expanding valency (evolution of 4-valent GARDASIL® maintaining the existing antigen types along with addition of 5 new types into 9-valent GARDASIL®9). It would be highly informative and accelerate the path to approval by better incorporating historical stability data to compare advanced Bayesian models alongside the traditional methods of reporting stability to regulatory agencies. Here we leverage historical data for more than 30 batches of this platform vaccine product via the hierarchical modelling approach.

Third, it is important that the interpretability of stability models has a robust physical basis and adheres to logical biopharmaceutical/physicochemical understanding even if findings are empirical in nature. Therefore, our models are of a hybrid form that leverage statistical mathematics in conjunction with an algorithm that mimics a biochemical reaction leveraging Arrhenius kinetics. Fourth, some large-molecule products have significant random error in analytical assays leading to high variability (“noisy data”), e.g., as is common for potency measurements. Our Bayesian model addresses this variability adequately (as will be further discussed). Finally, one cannot assume that specialized tailored data sets for the specific purpose of supporting predictive stability modelling will always be available in an industrial context, because there are often limitations in material quantities and confinements imposed by established standard operating procedure (SOP) test protocols.

Here we propose a model designed to work with a common set of ICH-guided temperature and time point conditions (for example, as historically conducted for GARDASIL®9). All data sets were derived from standard commercial stability data and not explicitly designed to support predictive stability. It is also acknowledged by the authors that there may be cases in the future where more factors/conditions may be needed to enhance the robustness of these models. To summarize, this work represents a significant advancement in the application of predictive stability for large molecules, in this case study of vaccines.

## Method

### Model selection & bayesian model benefits

A conventional stability modelling approach would create multiple, separate bespoke stability models; i.e., one model for each molecular type contained within this product. However, due to random variations in the data generated from the analytical assay release method, data extrapolation using simple linear regression techniques or Arrhenius-type equations (separately for each batch) led to inaccurate predictions and wide confidence intervals. Conversely, a hierarchical Bayesian model is a general solution that incorporates information from multiple batches and molecular types into a single model, which provides more stable predictions (as illustrated in Figure S3 in the **Supplementary Materials**) by leveraging historical batch data that is available to the end of shelf life or beyond.

In a hierarchical statistical model, parameters are grouped into layers (i.e., levels), allowing for the sharing of information across these layers. In this application, multiple model layers are used, including a “Batch Layer”, “Molecular Type Layer” and a “Package/Container Layer”. The “Batch Layer” accounts for stability data collected from different product-specific batches. The “Molecular Type Layer” accounts for stability of the batches of various analogous vaccine antigen sub-types contained within the same multivalent product (note that the 9 molecular types are identical across all the batches). The “Package/Container Layer” accounts for different corresponding configurations such as vial or pre-filled syringe. The hierarchical model structure allows for characterization of both the variation within, and between batches and across molecular types and containers, leading to more robust predictions with enhanced precision and accuracy than using a conventional Frequentist statistical approach. A detailed description of the model is included in the section ‘Model Specification’ in Supplementary Materials. The model is expandable as more information layers could be integrated in the future (e.g., other aspects related to the product, process, physicochemical attributes, computed molecular descriptors, etc.).

A comprehensive hierarchical approach leads to simplification by reducing long-term model maintenance and upkeep (1 unified model instead of 9 separate models) without sacrificing model performance. The model utilized in this study has been trained using a robust dataset comprising historical data from previous drug product batches of vials and syringes, which covers all the molecular types. These data consist of pre-existing analytical information derived from similar studies. By incorporating short-term experimental stability data from newly produced product batches, the model is capable of generating accurate stability predictions. Within the drug product of co-formulated molecular types, the biological activity of each individual type is assayed separately on a type-specific basis. For molecular types that behaved similarly in their stability profile within the combined product at the real-time and accelerated conditions across many batches, hierarchical model information can be shared across types to enhance model accuracy (i.e., when the stability performance of one antigen molecular type is representative of a group of other related types possessing similar stability loss mechanisms and trends over time).

Even in the absence of historical batch data serving as prior knowledge, Bayesian hierarchical modelling still affords benefits in its inherent data information-sharing capacity. Information data sharing across molecular types increases the accessible data density for prediction (on the empirical assumption that similar historical trends for different molecular types could be combined to predict the stability profiles of each separately; after all the molecular types are mixed inside the same container and subjected to the same macroenvironmental conditions). This increased information sharing is helpful because high-variability assays like potency can carry appreciable random analytical error which may be batch independent (this was the case for the current case study as will be discussed). Sharing of information prevents the likelihood of the model being misled by sporadic random (systemic) variance and outliers in assay values while becoming more likely to be able to identify the true long-term trend. Moreover, if there are changes occurring in the macroenvironment of the solution which would affect multiple components in the product then the model could be more likely to pick up such observations by comparing across all available data rather than only against singular isolated molecular components. Altogether, the Bayesian hierarchical model showed lower variability in the results and was less dependent on individual observations for making predictions. As more data are collected, the differences between a Frequentist model and the Bayesian model become smaller, as the two model estimates tend to converge to the same values, as shown in **Figure S5**.

An additional benefit of the Bayesian approach lies in assessing model confidence. A Bayesian model provides a coherent way to estimate credible intervals for parameter estimates and prediction intervals of the stability profiles, even for new batches that do not have long-term stability data to determine shelf-life. For example, the 95% credible interval band is readily compared to the stability specification limits in the Model Validation Section.

### Model description

The model, detailed in section ‘Model Specification’ in **Supplementary Materials**, was estimated using a Gibbs sampler, a Markov chain Monte Carlo algorithm that enables the generation of posterior samples for the model parameters, providing a way to generate credible intervals for the model parameters. In a similar manner, prediction intervals can be created for a given timepoint and batch in a coherent way within the model specification, and without needing a theoretical asymptotical calculation, which would often be needed in a Frequentist setting. The model was further assessed for accuracy of predictions by evaluating the impact of time points up to 36 months. The main attributes in the model are time, temperature, batch identifier, molecular types, package/container type, and measured assay values (potency). The Bayesian model’s predictions were validated by selecting target batches that statistically aligned at earlier timepoints with the historical training set, supporting that the forecasted stability of the target batches towards the end of shelf-life are accurate.

### Historical training data

Historical training data were gathered for thirty (30) batches of GARDASIL®9 at the designated storage temperature (5 °C) with 24–42 months long-term stability data, as well as stability data at accelerated storage conditions (25 °C and 37 °C). Data at 42 °C was also available under a different protocol but not incorporated for the predictions as it was deemed not necessary for predicting the storage condition, however it may be useful in the future to drive further accelerated assessment. Data for the shelf-life limiting attribute (potency) were utilized for model evaluation.

Bayesian sensitivity analysis can be applied to probe how a quantity of interest – here stability prediction to 36 months – changes with different selection of “prior” model parameters that define the distribution for slope and y-intercept, and parameters which affect the weighting for historical data. In a scenario where there are only a few historical data points, or when the historical data are sourced from a relatively dissimilar product, the prior can be selected to be less informative; that is less concentrated around estimated historical prior parameters, such that it is the newly acquired data that is primarily driving model inference. Conversely, in circumstances where the historical batches resemble the new batches or product (as in this example), more weight can be assigned to the historical data (such as the intercept and slope of stability profiles from historical batches). This allows for a more informed estimation of the model parameters for a current batch. Known intrinsic biases between historical and current data can be incorporated in the model; however, in the present examples there were no known biases that would adversely impact the model output (e.g., all data were analytically comparable).

Considering the substantial volume of data points (2160 measurements) utilized for model training, the priors were established in a non-informative manner. In other words, the large amount of historical data was sufficient to be relied upon as empirical evidence for making direct inferences about the model parameters without a need for introducing informative prior assumptions. Priors were chosen to be non-informative to allow the empirical data itself to more strongly influence the outcome of the analysis (with no bias from prior assumptions).

Simulations can be performed using historical data to inform how many observations are needed in the Bayesian model to gain accurate predictions. As mentioned previously, prediction intervals can be estimated sampling from the predictive distribution of the Bayesian model trained on historical data. As more simulated data is added to the analysis, prediction becomes more accurate and credible intervals have shorter length. This can be used to estimate how much data is needed for a target prediction width. Data from the thirty (30) batches were evaluated for prediction accuracy. While the authors elected to use all available data for the product for our primary analysis, we note that far fewer batches would also be suitable and elucidate recommendations on the minimum number of batches and time point data in the supporting information in Figs. 4, S3 and S4.

### Model validation

The Bayesian model was validated for each batch stability profile using potency measured at stability time points and temperatures as independent variables. This analysis verified that analytical assay variability was independent of the batch (i.e., overall variability was primarily related to the analytical assay). To verify the accuracy of these predictions with long-term data, the prediction coverage, which refers to the percentage of points within a predetermined prediction interval of level 1-alpha, is evaluated in a test set (typical choices of alpha include 0.01, 0.05, 0.1). In instances where the calibration of prediction intervals does not match the target, there are several possibilities to handle such discrepancies. One option is adjusting the scalar parameters’ prior distributions to either inflate or deflate prediction intervals to achieve the desired coverage. This methodology facilitates a rigorous examination of the Bayesian model’s performance against long-term data, allowing for any observed differences to be systematically addressed and accounted for in the prediction coverage and it is coherent with the definition of shelf-life as the lower 95% confidence bound at 36 months.

In this example, the prediction coverage was assessed using the aforementioned thirty (30) batches, yielding empirical coverage levels that closely matched the nominal coverage levels (i.e., with nominal coverage level 1-alpha the model well-covered the experimental data). Here, nominal coverage refers to the specified confidence level of an interval estimate which is intended to contain the true value with a given probability. Empirical coverage is the observed frequency with which these intervals actually contain the observed value when applied repeatedly to the data, reflecting the model’s practical performance. Data on a newly manufactured batch was used to prospectively verify the shelf-life prediction. Long-term model updates can leverage new stability time points to further improve predictions.

## Results

### Exploratory data analysis

We first outline an exploratory data analysis that was performed on historical stability data of GARDASIL®9 and then discuss the application of the Bayesian hierarchical model towards shelf-life prediction for this coformulated drug product composed of multiple molecular types.

**Fig. 1:**
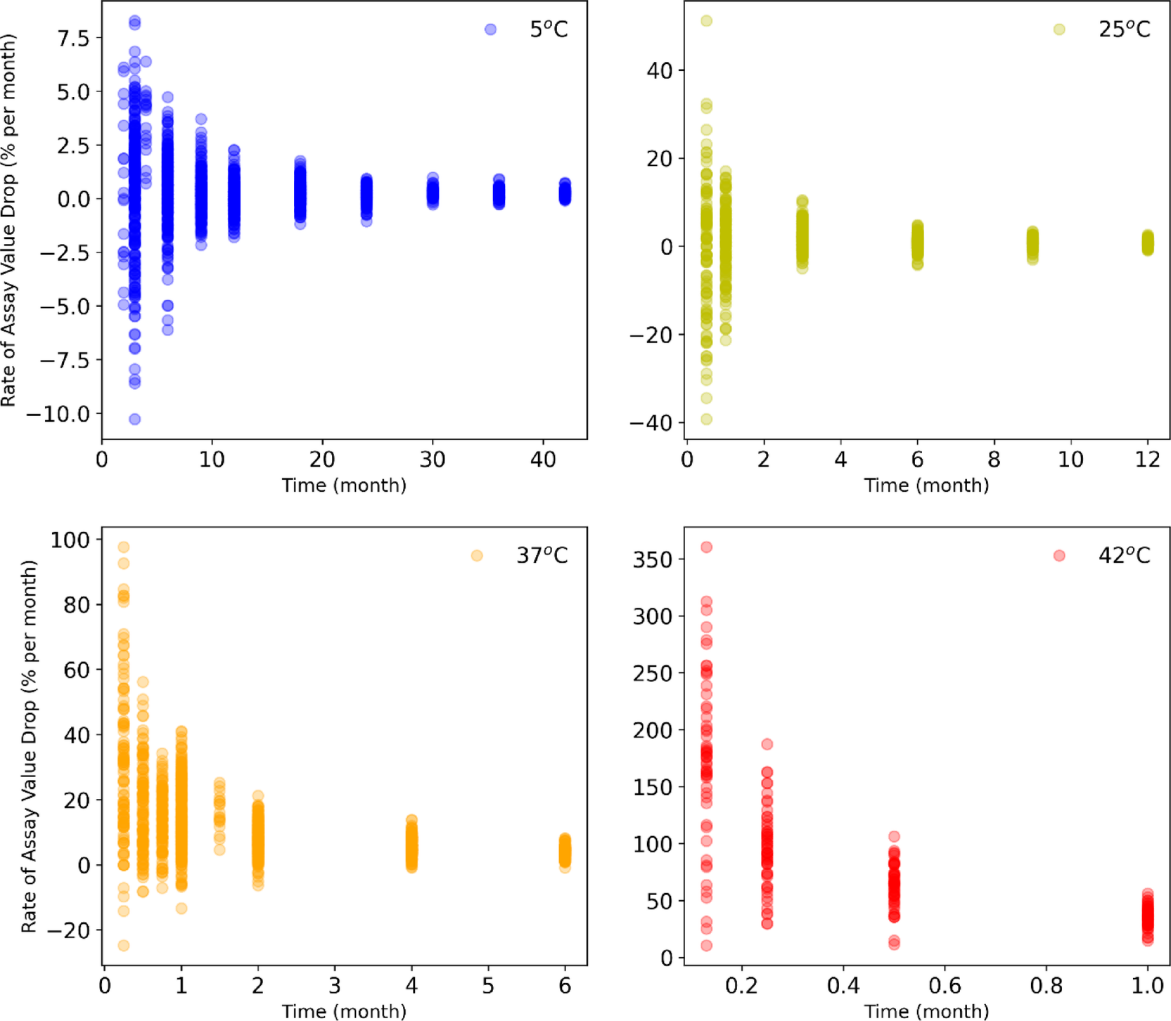
The rate of assay value drop indicated as a percent decrease on a per month basis in the stability indicating attribute (potency) for samples incubated at various temperature and time points for GARDASIL®9. All molecular types within the product are plotted across approximately 20 stability studies. The y-axis displays the average monthly percentage drop in the stability indicating attribute (i.e., the y-axis value is the net rate calculated as the change in the attribute at a given month relative to the initial time point divided by the number of total months the sample had been on stability).

As the first step, prior to model creation, exploratory data analysis was performed on the available stability data for GARDASIL®9 collected with an in vitro relative potency assay^15^ As shown in Fig. 1 historical trends in the stability indicating attribute (potency) with storage temperature and time were evaluated across all the molecular types contained within the product. The figure contains about 4,000 sampled data points across approximately twenty (20) stability studies. Intuitively, instability grew more pronounced with increasing temperature. The extent of the rate of change in the stability indicating attribute increased across the sequence from 5 °C →25 °C → 37 °C → 42 °C, suggesting a strong temperature dependence (i.e., an expected temperature dependence as a result of increasing collision frequency and enthalpically favored conformational states driving loss of available epitope as measured in potency).

An important additional observation is that the rates of attribute change were greater in the first few months whereas the change tapered off in subsequent months. Namely, the rate of decrease in the potency stability attribute becomes small after long time periods. This behavior implied that by capturing the changes observed in short-term experimental stability data for multiple temperatures (which constituted the bulk of the overall stability change), the model would accurately predict the long-term stability behavior. Moreover, even if the stability change does not drop off over a long stability time course duration for future batches, the model would not underestimate stability loss and rather would tend to overestimate instability risk. In other words, the exploratory data analysis supports the general principle of using early time points as a model basis for long-term prediction. A statistical analysis of variability in the data points suggested that the data variability was independent of the batch and approximately ≥ 80% of the variability was random and unrelated to the molecular type (e.g., related to analytical assay variability). Indeed, high random variability is common for potency assay measurements (this is why in some cases the measured potency appeared to exceed 100%). Negative rates in assay drop correspond to a measured increase in potency, likely attributable to assay variability.

### Model predictions

**Fig. 2:**
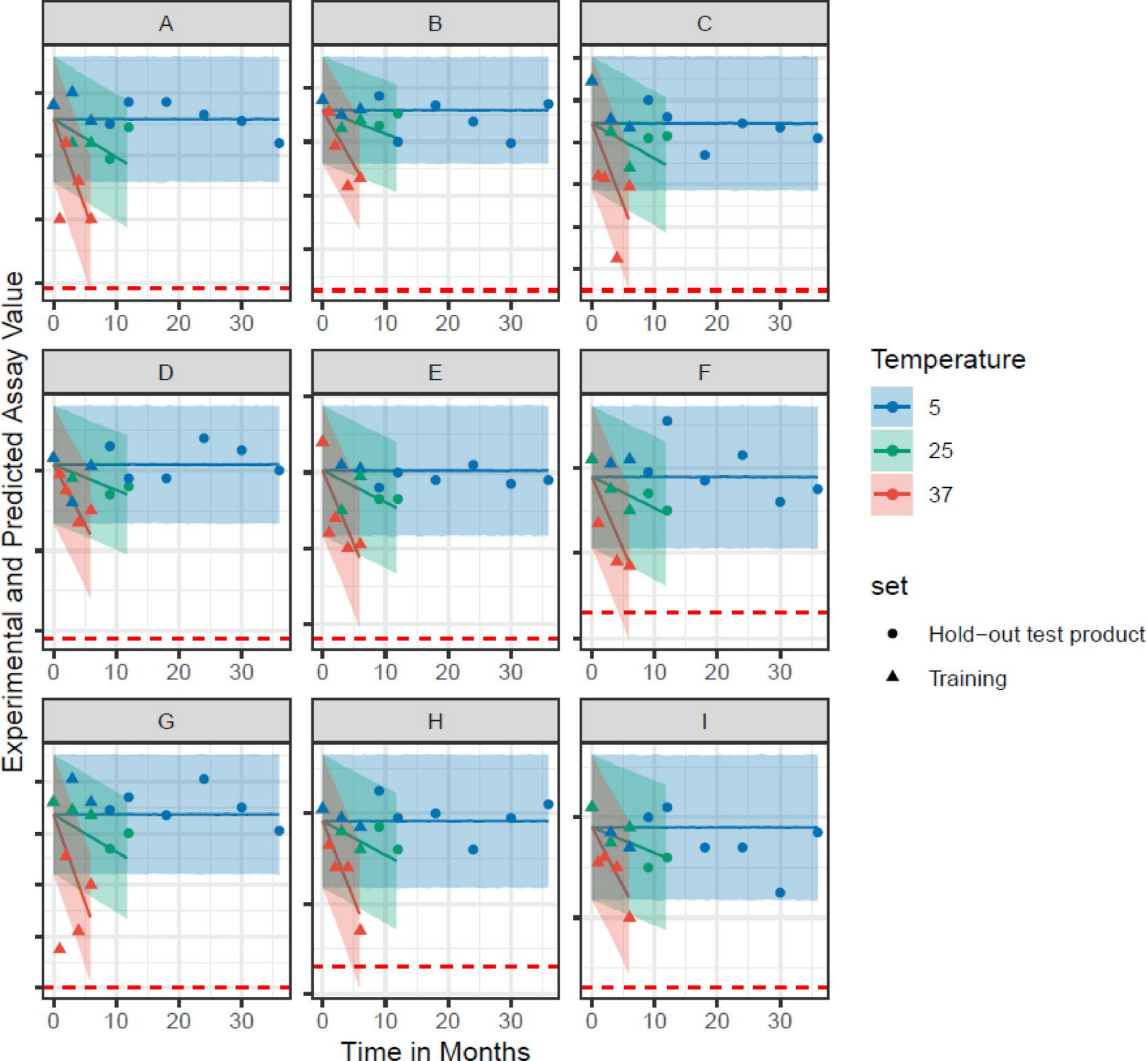
Hierarchical Bayesian model prediction for Training and Test sets for 9 molecular types (specific types were blinded, with the y-axis potency assay value normalized) contained within GARDASIL®9 (combined co-formulation vaccine product). 95% prediction intervals plotted as the banded regions. Three temperatures are shown at 5 °C (blue), 25 °C (green) and 37 °C (red). The training data (triangle points) and hold-out test product data (circles) are shown in the plot. The prediction interval for long-term storage at 5 °C is shown out to 36 months (the shelf-life of GARDASIL®9). The solid line in each of the intervals represents the average prediction for that given time value. The lower specification limits for the normalized potency are shown as red dotted lines.

The Bayesian model (details in section ‘Model Specification’ in Supplementary Materials), was estimated using data up to 6 months storage at 5 °C, 25 °C and 37 °C for all 30 batches, in order to assess model predictions for long-term 5 °C stability. For illustration, Fig. 2 shows examples corresponding to all antigen molecular types contained within a given batch. The model was trained on the first 6 months of data (triangles in the figure) and evaluated in relation to the measurements generated after 6 months (circles in the figure) (i.e., a “time-split” validation was performed by training the model on 0 to 6 months (0–6 M) data for all batches and predicting time points greater than 6 months). The Gibbs sampler (a Markov chain Monte Carlo algorithm) provides the estimation of parameter (slopes by temperature and y-intercept) distributions within the model, providing a way to generate prediction intervals (shaded regions) for the test dataset from product manufactured after a major process change.

**Fig. 3:**
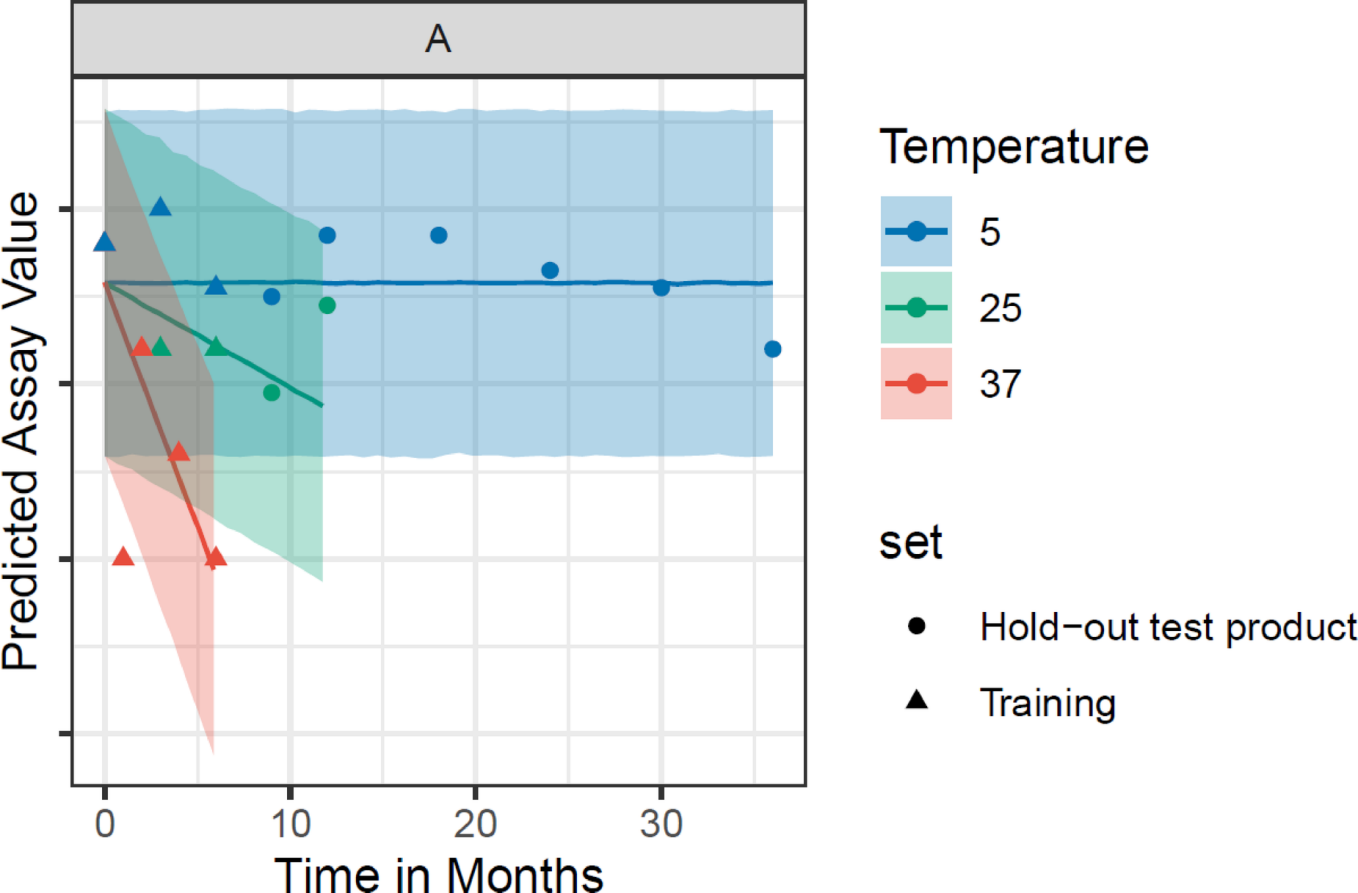
Hierarchical Bayesian model prediction for Training and Test sets for antigen molecular type A contained within GARDASIL®9 (combined co-formulation vaccine product). The model was trained on the first 6 months of data (triangles in the figure) and evaluated in relation to the measurements generated after 6 months (circles in the figure) (i.e., a “time-split” validation was performed by training the model on 0 to 6 months (0–6 M) data for all batches and predicting time points greater than 6 months).

The figure demonstrates that using a hierarchical Bayesian model for a co-formulated drug product, parameters defining the stability profile over time for one molecular type can help inform and predict other molecular types in tandem (in this example of 9 antigen molecular entities). Figure 3 shows an example zoom-in of the model for a test manufactured batch for molecular entity **A** along with the prediction intervals (shaded regions) from Fig. 2. As shown in Fig. 2, the predictions and associated intervals at 5 °C and 25 °C do not intersect the attribute stability limit (red dotted line) even after extended periods of time. Figure S1 (Full Dataset) demonstrates that the Bayesian Hierarchical model is calibrated, as evidenced by the alignment of Empirical and Nominal Coverage at corresponding alpha levels, indicating the model’s reliable uncertainty estimates. Figure S2 shows that the model performs well in both training and test sets across all temperatures, with the predictions closely aligning with the actual assay values. The Bayesian model can thus be employed to predict shelf life of a new batch manufactured after a process or other change, by jointly building a model across different historical, representative batches and molecular types, using early time point data of up to 6 months and extrapolating out for a 36 month prediction. The model is actively applied with ongoing verification of the new or otherwise representative batches to confirm shelf-life with real-time data prospectively (i.e., newly collected data points fall within the prediction intervals). We acknowledge that while the model appears to have great applicability to a multivalent vaccine, it is important to continue to assess suitability and validation of the model particularly for future use cases and to consider its application as one component of a comprehensive scientific risk-based decision framework.

### Model comparisons and data requirements

A set of simulations was designed to assess the Bayesian model performance over a different set of scenarios and compare it to (1) a collection of simple linear models trained on each batch separately and (2) a full mixed effect model containing random effects for batch number, an interaction term between time and temperature and a fixed effect for package/container. The different model performances were evaluated using coverage at the 95% nominal level on the hold-out data set at 5 C. Table 1 contains a summary of the simulations. The simulation experiments were conducted under three distinct settings: full dataset, syringe-only, and vial-only. The full dataset comprised of 30 batches, while the syringe-only and vial-only settings included 17 and 13 batches, respectively. For each setting, model estimation was performed using various numbers of time points (4, 6, 9, and 12) across a range of training set batch sizes: 5, 10, 15, 20, and 30 for the full dataset, and 5 and 10 for the syringe-only and vial-only datasets. In each simulation, a subset of batches was sampled and used to train the models. A total of 50 simulations were run for each scenario to evaluate the statistical model’s performance.

**Fig. 4:**
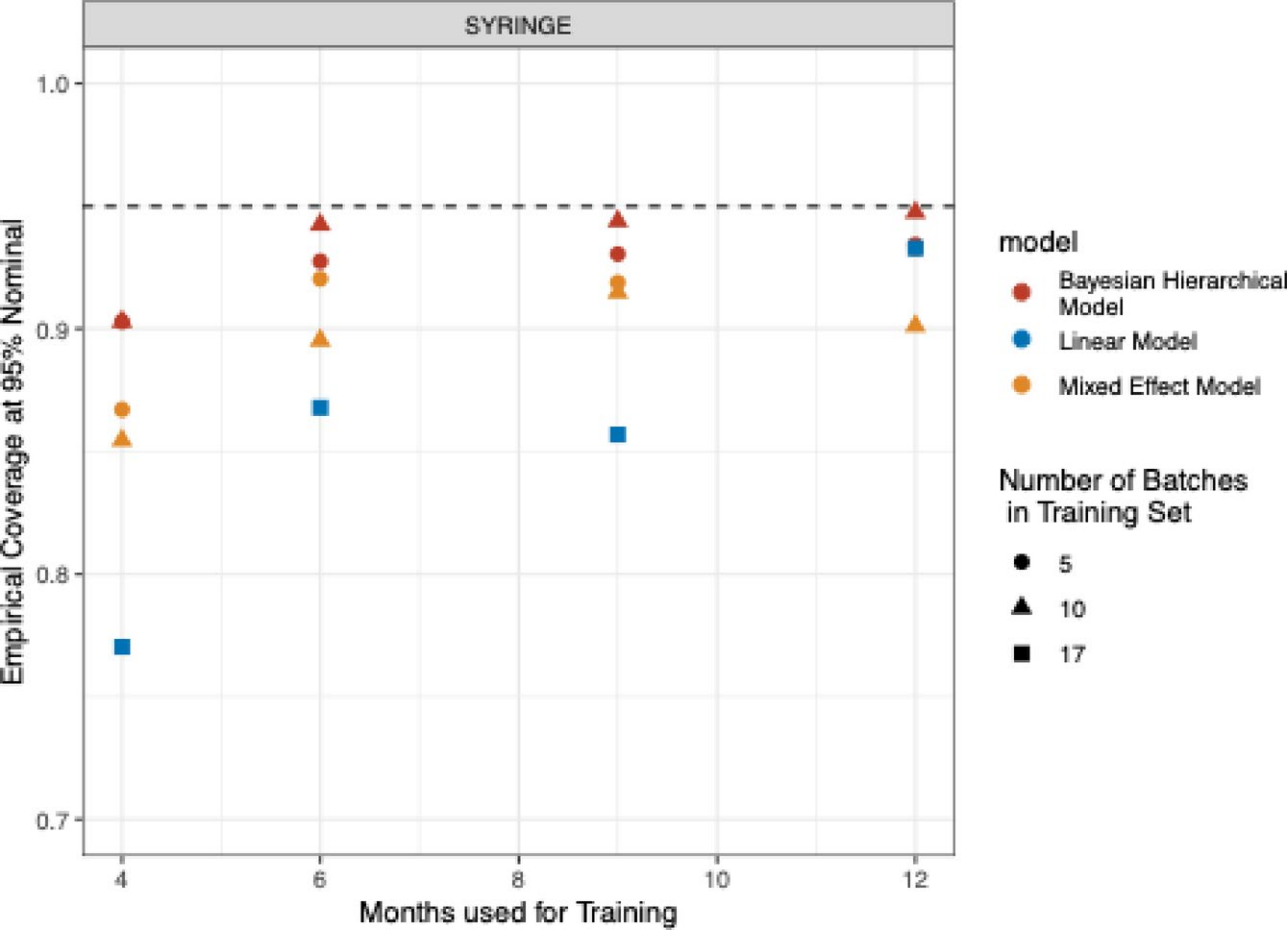
Empirical coverage at 95% nominal for Bayesian Hierarchical model, linear model, and mixed effects model as a function of months used for training and number of batches in the training set for syringe for the in-vitro relative potency assay. Different shapes represent the number of batches in the training set: circles (5), triangles (10), and squares (17). The dashed line indicates the nominal coverage of 95%.

The results in Figure S3 highlight the superior performance of the Bayesian Hierarchical Model compared to both the Linear and Mixed Effects Models, particularly when using fewer batches and shorter durations of stability data. The Bayesian model consistently achieves empirical coverage close to the nominal 95% level with as little as 6 months of data and 5 batches, while the Linear Model and Mixed Effect Model require a larger dataset to perform comparably. Figure 4 presents the empirical coverage at 95% nominal for the syringe container for the three models and with different data quantities. Similar data for the vial container is shown in Figure S4. The Bayesian Hierarchical Model (red) generally achieves higher and more consistent coverage near the nominal 95%, particularly with larger batch sizes and longer training durations. The Linear Model (blue) exhibits lower coverage overall, especially in the syringe setting, where the empirical coverage drops below 90% for several scenarios. The Mixed Effects Model (orange) shows intermediate performance, with better coverage than the Linear Model but generally not reaching the robustness of the Bayesian approach. Overall, the Bayesian model demonstrates superior and more stable coverage across the various conditions compared to the Linear and Mixed Effects Models, especially as the training data size increases.

In Figure S5 it is demonstrated that the Bayesian Hierarchical Model consistently achieves lower mean squared error (MSE) than the Linear Model across all HPV types, regardless of the number of months used for training. The MSE for the Bayesian model remains low even with shorter training durations, while the Linear Model exhibits significantly higher errors, especially when trained with less data.

**Table 1:** Summary of simulation experiments.

Simulation Setting	Total Batches	Number of batches in training set	Time points used in model estimation	Total Simulations
Full Dataset	30	5, 10, 15, 20, 30	4, 6, 9, 12	50
Syringe Only	17	5, 10	4, 6, 9, 12	50
Vial Only	13	5, 10	4, 6, 9, 12	50

## Conclusions

The Bayesian hierarchical model allows for more comprehensive, robust, precise and coherent predictions, by effectively combining current data with prior platform information. As illustrated by the case study example of GARDASIL®9, the Bayesian hierarchical model is especially useful for forecasting long-term stability out to end of shelf life of newly manufactured representative batches based on short-term data and to establish shelf-life. Additionally, the model can help inform on impact of process, container-closure, and other types of changes. It can also be beneficial in clinical studies for setting longer shelf life of clinical phase materials when longer-term real-time stability remains unavailable, while outperforming alternative approaches. It has particular utility for complex products such as those composed of multiple molecular entities, those examining analytical assays possessing large random variability in assay data, and for products with multiple container types like vial and syringe. An important aspect of long-term model verification is the use of independent experiments; to this end, we have actively monitored new or otherwise representative batches with the model and confirmed that its performance aligns with the predictions.

An additional benefit is that once established, this model provides scientific insights to inform the development of future products of the same molecular class. Indeed, it is highly common for new drug substances to be added in the form of combination biopharmaceutical products or in increased valency of vaccines to tackle different viral strains. For new analogous molecule types, e.g. similar types targeting other strains of the same group of viruses, the model outputs can be used to affirm that product stability trends follow expected behavior based on learned information from the existing types in conjunction with a smaller quantity of long-term experimental stability data from the new types. This aspect is currently under investigation. Testing for new analogous molecule types and for assessing major changes to manufacturing process and manufacturing site is ongoing.

We are also actively investigating the utility of the model architecture for different types of vaccines (viral, inactivated, recombinant protein sub-unit, and conjugates) as well as multiple biotherapeutics where its performance has shown success given limited early stage data. We have found that for products with less availability of stability data the model can still perform well or at least equivalently to a more conventional statistical or kinetic modeling framework. Application to novel modalities and adjuvants may also be possible and this is a topic of investigation. In the **Supplementary Materials**, we show the generalizability of our model to estimate the antigen content of another Merck vaccine, which has been described and analyzed in (31). In particular, we illustrate the advantage of employing a hierarchical model specification, which allows to achieve accurate predictions utilizing only a portion of the data to train the model.

## Electronic supplementary material

Below is the link to the electronic supplementary material.

Supplementary Material 1

## Data Availability

The datasets used and/or analyzed during the current study available from the corresponding author on reasonable request.

## References

[1] Joura, E. A. et al. A 9-Valent HPV vaccine against infection and intraepithelial neoplasia in women. *New. Engl. J. Med.* **372** (8), 711–723. 10.1056/NEJMoa1405044 (2015). 25693011 10.1056/NEJMoa1405044

[2] Keerti Halemani, S. H. J. Advancements in HPV vaccination for cervical Cancer prevention: A comprehensive review. *Saudi J. Nurs. Health Care*. **7** (5), 122–123 (2024).

[3] Immunization supply chain and logistics: a neglected but essential system for national immunization programmes: a call-to-action for national programmes and the global community; WHO/IVB/14.05; World Health Organization, Geneva, 2014. https://www.who.int/publications/i/item/WHO-IVB-14.05

[4] Rosalik, K., Tarney, C. & Han, J. Human papilloma virus vaccination. *Viruses-Basel*. 10.3390/v13061091 (2021). 10.3390/v13061091 PMC8228159 34201028

[5] Comes, T., Bergtora Sandvik, K. & Van de Walle, B. Cold chains, interrupted. *J. Humanitarian Logistics Supply Chain Manage.* **8** (1), 49–69. 10.1108/JHLSCM-03-2017-0006 (2018). (acccessed 2024/07/08).

[6] McGoldrick, M. et al. How to accelerate the supply of vaccines to all populations worldwide? Part II: Initial industry lessons learned and detailed technical reflections leveraging the COVID-19 situation. *Vaccine* **40** (9), 1223–1230. 10.1016/j.vaccine.2021.12.038 (2022). From NLM Medline. 35180994 10.1016/j.vaccine.2021.12.038 PMC8846337

[7] McMahon, M. E. et al. Considerations for updates to ICH Q1 and Q5C stability guidelines: embracing current technology and risk assessment strategies. *AAPS J.* **23** (6), 107. 10.1208/s12248-021-00641-6 (2021). 34529169 10.1208/s12248-021-00641-6

[8] Concept paper on the revision of ICH guidelines Q1 and Q5C (No. *Final 2022/1114)* (International Council for Harmonisation of Technical Requirements for Pharmaceuticals for Human Use, 2022).

[9] Jain, T. et al. Biophysical properties of the clinical-stage antibody landscape. *P Natl. Acad. Sci. USA*. **114** (5), 944–949. 10.1073/pnas.1616408114 (2017). 10.1073/pnas.1616408114 PMC5293111 28096333

[10] McMahon, M. et al. Utilization of risk-based predictive stability within regulatory submissions; industry’s experience. *AAPS Open.* **6** (1), 1. 10.1186/s41120-020-00034-7 (2020).

[11] Ramin, E. et al. Accelerating vaccine manufacturing development through model-based approaches: current advances and future opportunities. *Curr. Opin. Chem. Eng.*10.1016/j.coche.2023.100998 (2024).

[12] Li, M. Y. et al. Lyophilization process optimization and molecular dynamics simulation of mRNA-LNPs for SARS-CoV-2 vaccine. *Npj Vaccines*. 10.1038/s41541-023-00732-9 (2023). 37813912 10.1038/s41541-023-00732-9 PMC10562438

[13] Thiagarajan, G., Semple, A., James, J. K., Cheung, J. K. & Shameem, M. A comparison of biophysical characterization techniques in predicting monoclonal antibody stability. *Mabs-Austin* **8** (6), 1088–1097. 10.1080/19420862.2016.1189048 (2016). 10.1080/19420862.2016.1189048 PMC5037989 27210456

[14] Bennett, A. et al. Thermal stability as a determinant of AAV serotype identity. *Mol. Ther-Meth Clin. D*. **6**, 171–182. 10.1016/j.omtm.2017.07.003 (2017). 10.1016/j.omtm.2017.07.003 PMC5552060 28828392

[15] Shank-Retzlaff, M. L. et al. Evaluation of the thermal stability of Gardasil®. *Hum. Vaccines*. **2** (4), 147–154. 10.4161/hv.2.4.2989 (2006). 10.4161/hv.2.4.2989 17012891

[16] Gao, F., Lockyer, K., Burkin, K., Crane, D. T. & Bolgiano, B. A physico-chemical assessment of the thermal stability of Pneumococcal conjugate vaccine components. *Hum. Vacc Immunother*. **10** (9), 2744–2753. 10.4161/hv.29696 (2014). 10.4161/hv.29696 PMC4977451 25483488

[17] Zhang, S. S. et al. Prediction of long-term polysorbate degradation according to short-term degradation kinetics. *Mabs-Austin*. 10.1080/19420862.2023.2232486 (2023). 10.1080/19420862.2023.2232486 PMC10332185 37415319

[18] Smith, W. J. et al. Impact of aluminum adjuvants on the stability of Pneumococcal polysaccharide-protein conjugate vaccines. *Vaccine* **41** (35), 5113–5125. 10.1016/j.vaccine.2023.05.059 (2023). 37321893 10.1016/j.vaccine.2023.05.059

[19] Heads, J. T., Kelm, S., Tyson, K. & Lawson, A. D. G. A computational method for predicting the aggregation propensity of IgG1 and IgG4(P) mAbs in common storage buffers. *Mabs-Austin*. 10.1080/19420862.2022.2138092 (2022). 10.1080/19420862.2022.2138092 PMC9704409 36418193

[20] Bunc, M., Hadzi, S., Graf, C., Boncina, M. & Lah, J. Aggregation time machine: A platform for the prediction and optimization of Long-Term antibody stability using Short-Term kinetic analysis. *J. Med. Chem.* **65** (3), 2623–2632. 10.1021/acs.jmedchem.1c02010 (2022). 35090111 10.1021/acs.jmedchem.1c02010 PMC8842250

[21] Waight, A. B. et al. A machine learning strategy for the identification of key descriptors and prediction models for IgG monoclonal antibody developability properties. *Mabs-Austin*. 10.1080/19420862.2023.2248671 (2023). 10.1080/19420862.2023.2248671 PMC10448975 37610144

[22] Bailly, M. et al. Predicting Antibody Developability Profiles Through Early Stage Discovery Screening. *Mabs-Austin *. (2020). 10.1080/19420862.2020.1743053 10.1080/19420862.2020.1743053 PMC7153844 32249670

[23] Gruia, F., Du, J. L., Santacroce, P. V., Remmele, R. L. & Bee, J. S. Technical decision making with higher order structure data: impact of a formulation change on the higher order structure and stability of a mAb. *J. Pharm. Sci-Us*. **104** (4), 1539–1542. 10.1002/jps.24158 (2015). 10.1002/jps.24158 25270279

[24] Bajoria, S. et al. Antigen-adjuvant interactions, stability, and immunogenicity profiles of a SARS-CoV-2 receptor-binding domain (RBD) antigen formulated with aluminum salt and CpG adjuvants. *Hum. Vacc Immunother*. 10.1080/21645515.2022.2079346 (2022). 10.1080/21645515.2022.2079346 PMC9621007 35666264

[25] Weiss, W. F., Young, T. M., Roberts, C. J. & Principles Approaches, and challenges for predicting protein aggregation rates and shelf life. *J. Pharm. Sci-Us*. **98** (4), 1246–1277. 10.1002/jps.21521 (2009). 10.1002/jps.21521 18683878

[26] Bannigan, P. et al. Machine learning directed drug formulation development. *Adv. Drug Deliver Rev.*10.1016/j.addr.2021.05.016 (2021). 10.1016/j.addr.2021.05.016 34019959

[27] Narayanan, H. et al. Design of biopharmaceutical formulations accelerated by machine learning. *Mol. Pharmaceut*. **18** (10), 3843–3853. 10.1021/acs.molpharmaceut.1c00469 (2021). 10.1021/acs.molpharmaceut.1c00469 34519511

[28] Rathore, A. S., Nikita, S., Thakur, G. & Mishra, S. Artificial intelligence and machine learning applications in biopharmaceutical manufacturing. *Trends Biotechnol.* **41** (4), 497–510. 10.1016/j.tibtech.2022.08.007 (2023). 36117026 10.1016/j.tibtech.2022.08.007

[29] Maharjan, R. et al. Recent trends and perspectives of artificial intelligence-based machine learning from discovery to manufacturing in biopharmaceutical industry. *J. Pharm. Invest.* **53** (6), 803–826. 10.1007/s40005-023-00637-8 (2023).

[30] Kuzman, D. et al. Long-term stability predictions of therapeutic monoclonal antibodies in solution using Arrhenius-based kinetics. *Sci. Rep-Uk*. 10.1038/s41598-021-99875-9 (2021). 10.1038/s41598-021-99875-9 PMC8519954 34654882

[31] Campa, C. et al. Use of stability modeling to support accelerated vaccine development and supply. *Vaccines-Basel*. 10.3390/vaccines9101114 (2021). 34696222 10.3390/vaccines9101114 PMC8539070

[32] Huelsmeyer, M. et al. A universal tool for stability predictions of biotherapeutics, vaccines and in vitro diagnostic products. *Sci. Rep-Uk*. 10.1038/s41598-023-35870-6 (2023). 10.1038/s41598-023-35870-6 PMC10284933 37344503

[33] Cordero, M., Meinfelder, F. & Eilert, T. A. Modern approach to stability studies via bayesian linear mixed models incorporating auxiliary effects. *J. Pharm. Sci.*10.1016/j.xphs.2024.02.020 (2024). 38417792 10.1016/j.xphs.2024.02.020

