# Supplementary material for: Bayesian hierarchical model predicts biopharmaceutical stability indicators and shelf life with application to multivalent human papillomavirus vaccine
Source: Sci Rep. 2025 May 19;15:17333. doi: 10.1038/s41598-025-99458-y (PMC12089594; doi:10.1038/s41598-025-99458-y)
Supplement: Supplementary file 1 — Supplementary Material 1 [file 41598_2025_99458_MOESM1_ESM.docx]

**SUPPLEMENTARY MATERIALS**

**Model Specification**

This model, following the specification in (1), introduces a hierarchical structure for assay values across different temperature settings, packaging types, lots, and HPV types, as illustrsated below:

$y_{i}= \alpha*{pkg}_{i}+ \alpha_{{type}_{i},{lot}_{i}}-k_{{type}_{i}, {lot}_{i},{pkg}_{i}}*\exp\left( \frac{Exp(E^{*})}{R} *\left( \frac{1}{{Temp}_{ref}}-\frac{1}{{Temp}_{i}} \right) \right)*{time}_{i}+ \varepsilon_{i}$,

$\varepsilon_{i}\sim N(0, \sigma_{{type}_{i},{pkg}_{i}}^{2}$).

**Data:**

- **Temperature (Temp)** in Kelvin and **time** are provided as predictors.
- **Package (pkg)** refers to a categorical variable with two levels (vial and syringe).
- **lot** represents the different manufacturing lots of the product.
- **HPV Type** categorizes the assay data according to different types of the virus.
- The response variable, **y**, represents the observed assay values.

**Constants:**

- **R = 1.987** Universal Gas Constant (cal⋅K−1⋅mol−1)
- $\mathbf{Temp}_{\mathbf{ref}}$ **= 298 (^o^K)**, which corresponds to ${25}^{o}C$

**Parameters:**

1. $k_{{type}_{i}, {lot}_{i},{pkg}_{i}}$**:** The rate constant matrix, defined for each HPV type, package and lot. It is drawn from a normal distribution with mean $k_{{type}_{i}}$, which is also drawn from a distribution that is non-informative.
2. $\alpha_{{type}_{i},{lot}_{i}}$**:** The intercept parameter which is varying by HPV type and lot. It is drawn from a normal distribution with mean $\alpha_{{type}_{i}}$, which is also drawn from a hyperprior distribution that is non-informative.
3. $\alpha$**:** This parameter reflects the effect of the package type, drawn from a normal distribution.
4. $E^{*}$**:** Represents the logarithm of the activation energy, drawn from a non-informative normal distribution.
5. $\sigma_{{type}_{i},{pkg}_{i}}^{2}$**:** A matrix representing the residual variability across HPV types and package types, modeled with a hierarchical inverse gamma distribution.

**Supplementary Figures**

**Figure S1.** Calibration plot comparing nominal coverage against empirical coverage at several *alpha* levels for the Bayesian Hierarchical model within the full dataset setting of **Table 2** (30 batches and 6 months of training data). The empirical coverage is estimated on the data points collected after 6 months and is defined at a given *1-alpha* level as the proportion of observations falling within the prediction interval of level *1-alpha*. This model is appropriately calibrated, as evidenced by the alignment of empirical and nominal coverage at corresponding alpha levels, indicating the model's reliable uncertainty estimates.


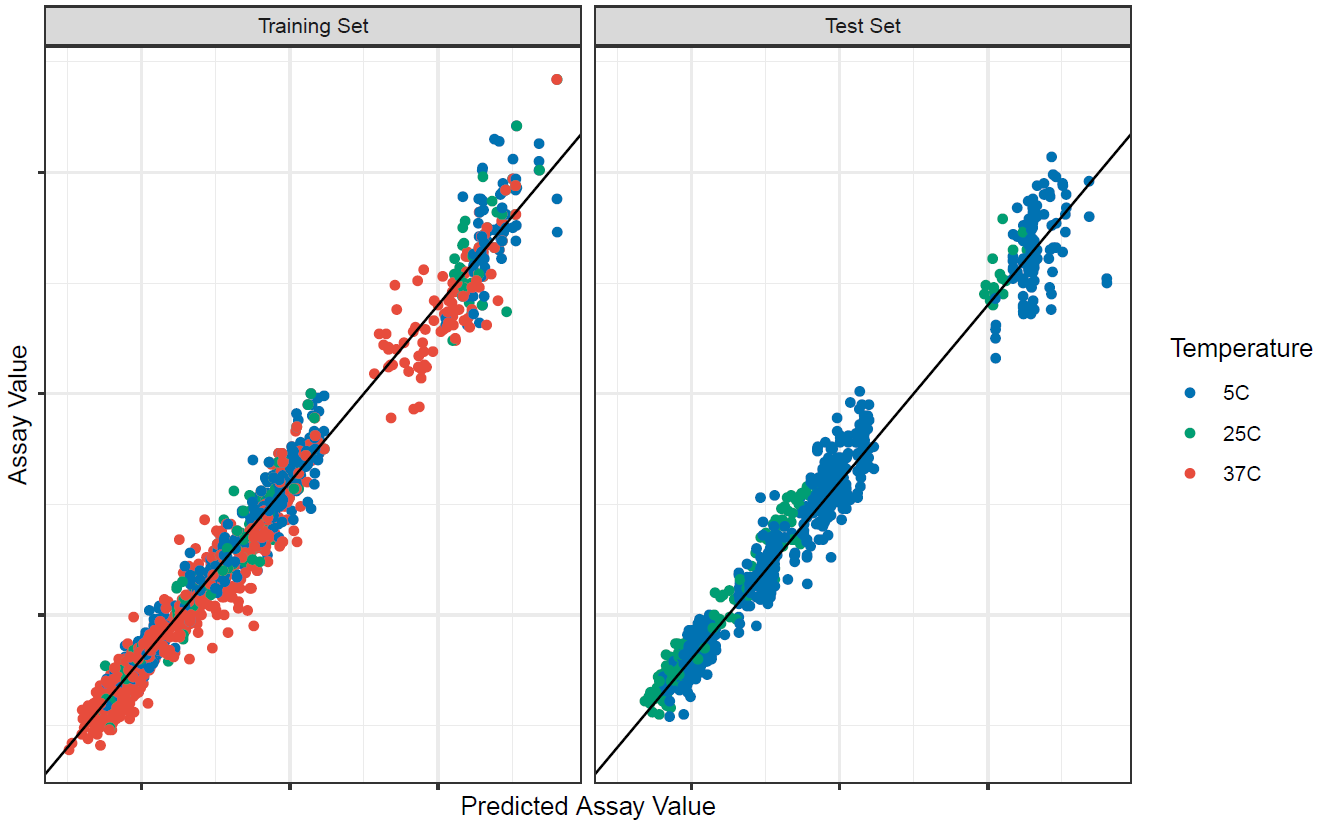


**Figure S2.**  Scatterplots comparing the predicted assay values (potency) across all HPV types with the Bayesian Hierarchical model to the observed assay values for the training (left) and test (right) sets at three temperatures: 5°C, 25°C, and 37°C. The diagonal line represents the line of perfect concordance. The model was trained within the full dataset setting of **Table 2** with 30 batches and 6 months of training data. Note that the specific assay value has been blinded.


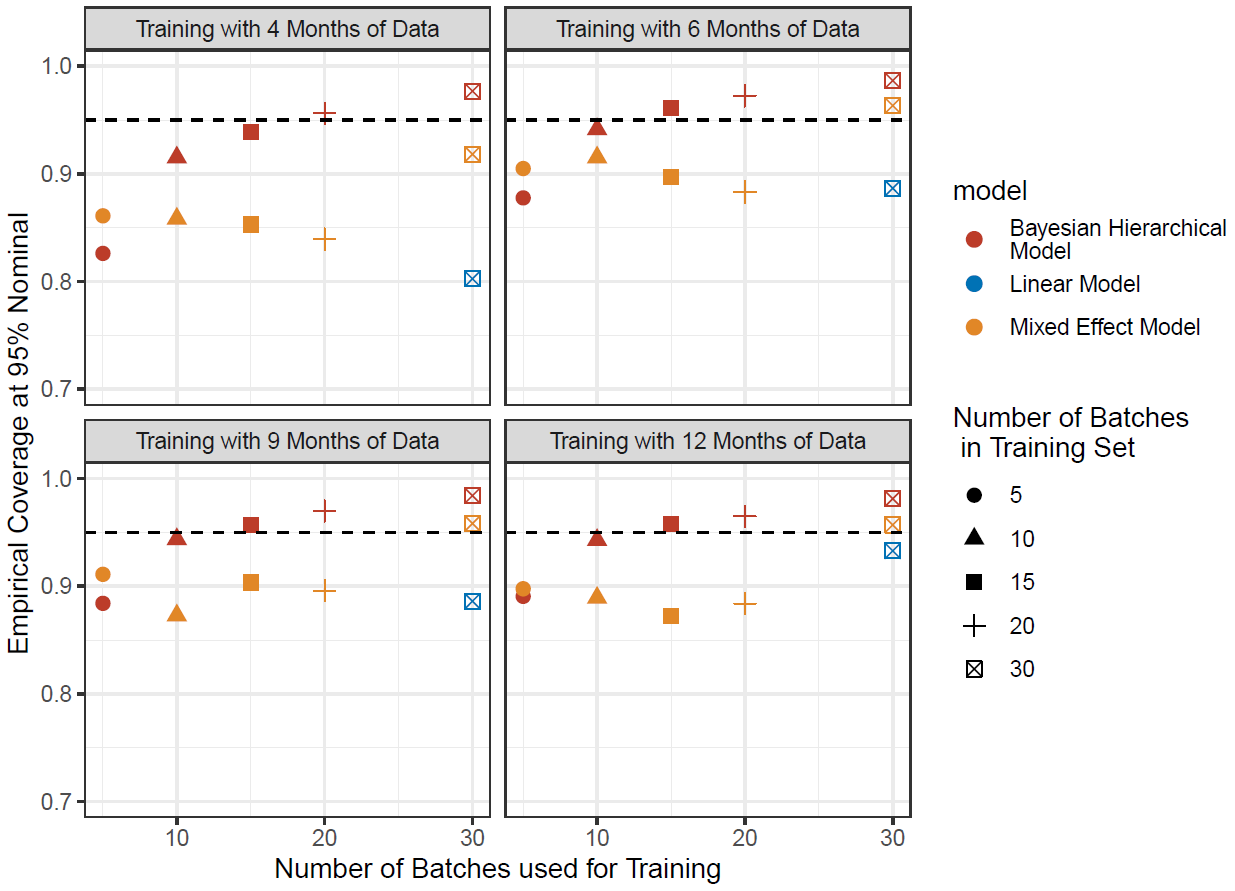


**Figure S3**. The amount of experimental data needed for effective predictions is probed (minimum number of batches and minimum duration of the short-term stability time course). Comparison of different models trained from varying the number of months of stability data (only 4, 6, 9 or 12 months of data) and number of batches, showing empirical coverage at 95% nominal level in the test set. Results are averaged across 50 simulations wherein different subsets of batches (selected out of 30 total) were used for training. These comparisons are created for the high-complexity large-molecule case study in this manuscript involving a high-variability potency assay and leveraging only non-informative priors in the Bayesian model and should not be taken as universal recommendations. For stability forecasting in a research and development setting, the Bayesian hierarchical model provides directional guidance that is useful with a few months of data from a few batches. For stability forecasting when needing to achieve a level of correct model calibration as would be used for commercial products, the Bayesian hierarchical model outperforms conventional batch-specific linear model (LM) and mixed effect model (MM) in needing only 6 months of data from 10 total batches whereas LM only performs adequately after training with 6 months of data and the full dataset. The empirical coverage at 95% nominal level is defined as the percentage of data points falling within a 95% prediction interval.

**Figure S4** Empirical coverage at 95% nominal for Bayesian Hierarchical model, linear model, and mixed effects model as a function of months used for training and number of batches in the training set for vial package. Different shapes represent the number of batches in the training set: circles (5), triangles (10), and squares (13). The dashed line indicates the nominal coverage of 95%.

**
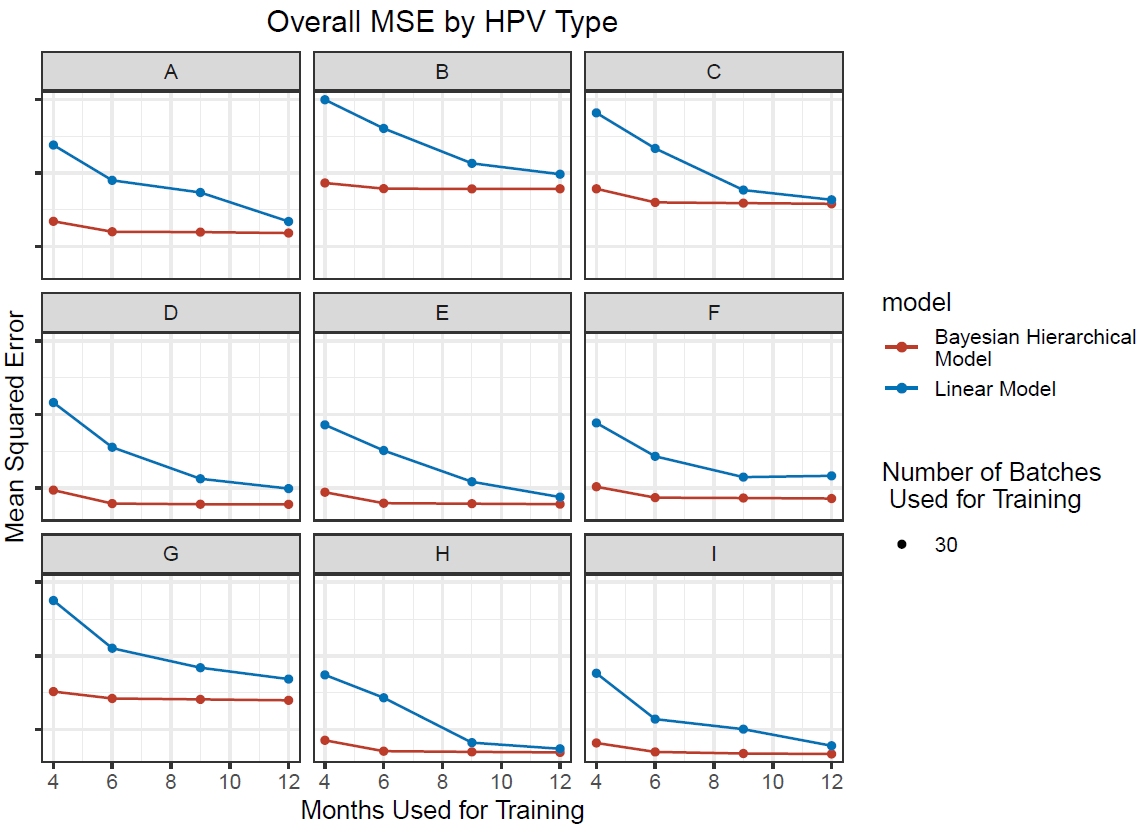
**

**Figure S5**. Mean squared error (MSE) by HPV type (specific types were blinded and match with **Figure 2** in the main text) for the Bayesian Hierarchical Model (red) and Linear Model (blue) as a function of months used for training (4, 6, 8, and 12 months) using 30 batches. Each panel represents a different HPV type, showing the decrease in error as the duration of training data increases.

**ELISA Antigen Content Estimation**

The Bayesian Hierarchical model detailed in this work has wider applicability beyond the specific GARDASIL®9 dataset, and can be adapted for larger/smaller datasets or different operational vaccine types (viral, inactivated, recombinant protein sub-unit of various numbers of serotypes, and conjugates) without needing significant modification. In this section, we show an application of the Bayesian hierarchical model to estimate the antigen content of another Merck & Co., Inc. vaccine, which has been described and analyzed in (2). Nineteen batches of data collected using the ELISA method at temperatures 5°C, 25°C and 37°C are considered (see **Figure 3** of (2)). A modified version of the model is estimated for this dataset:

$y_{i}= \alpha-k_{{lot}_{i}}*\exp\left( \frac{Exp(E^{*})}{R} *\left( \frac{1}{{Temp}_{ref}}-\frac{1}{{Temp}_{i}} \right) \right)*{time}_{i}+ \varepsilon_{i}$,

$\varepsilon_{i}\sim N(0, \sigma^{2}$),

where we assume a hierarchical specification for the parameter $k_{{lot}_{i}}$, which is drawn from a normal distribution with mean $k_{0}$, also drawn from a normal distribution, set as non-informative. We estimate the model using only five lots (Lot 1 through 5) and data points collected before month 6. We model the antigen content on the logarithmic scale and exponentiate back to predict on the original scale of the measurements. **Figure S6** contains the prediction results on the 19 batches. The hierarchical Bayesian model is able to accurately predict on hold-out long-term storage data for the training lots (Lot 1 through 5) and generalize to lots not used for prediction (Lot 6 through 19). This example further demonstrates the advantage of employing a hierarchical model specification, enabling us to achieve predictions that are comparable to those in (1), while utilizing only a portion of the data to train the model.


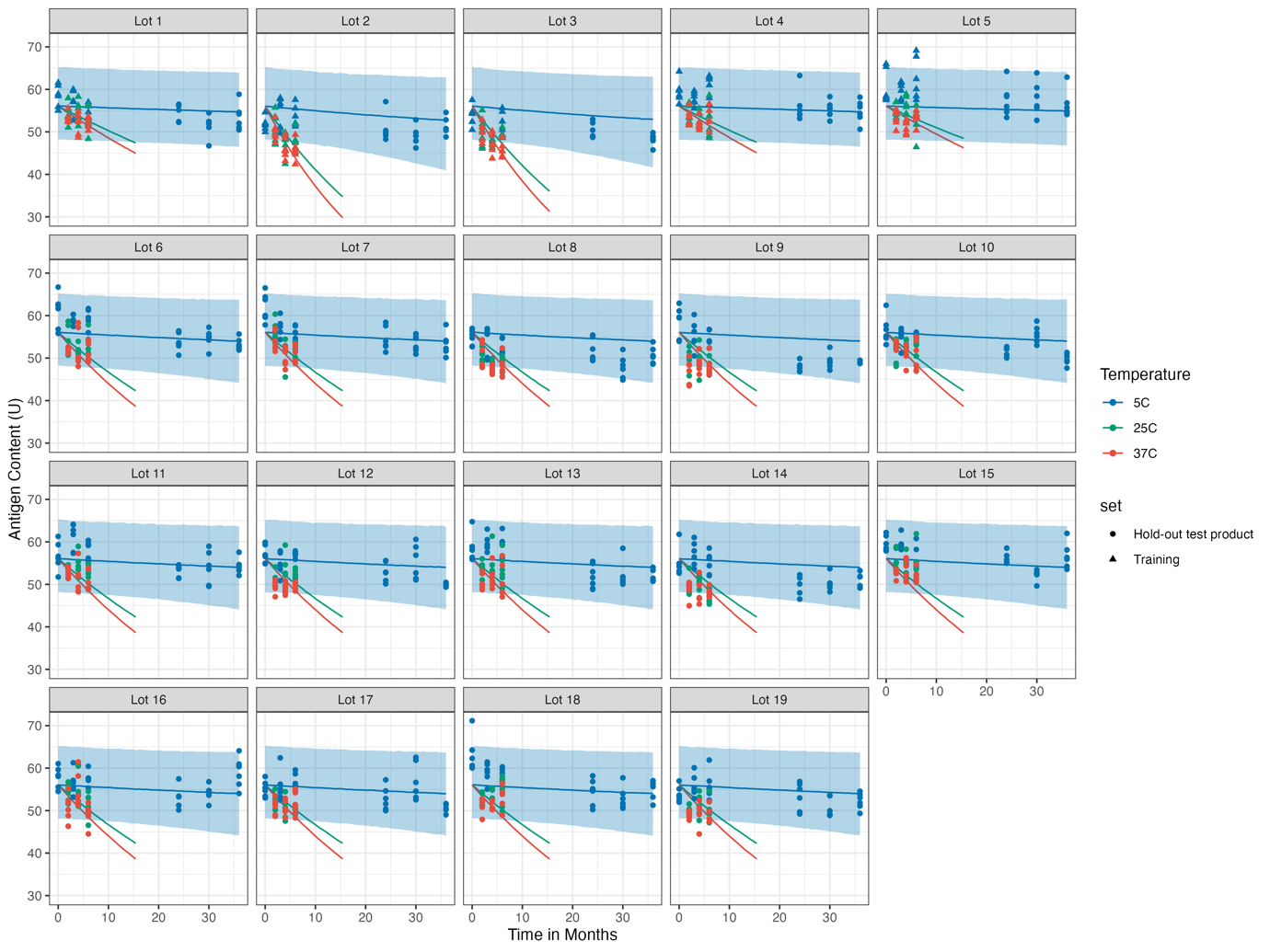


**Figure S6**. Hierarchical Bayesian model prediction for training and test sets for 19 batches. Three temperatures are shown at 5°C (blue), 25°C (green) and 37°C (red). The training data (triangle points) and hold-out test product data (circles) are shown in the plot. The model was trained only on the first 6 months of data of Lots 1 through 5 (first row in the figure). The 95% prediction interval for long-term storage at 5°C is shown out to 36 months.

**REFERENCES**

(1) King, Shang-Ying P., Min-Shya Kung, and Ho-Leung Fung. "Statistical prediction of drug stability based on nonlinear parameter estimation." Journal of pharmaceutical sciences 73.5 (1984): 657-662.

(2) Campa, C.; Pronce, T.; Paludi, M.; Weusten, J.; Conway, L.; Savery, J.; Richards, C.; Clenet, D. Use of Stability Modeling to Support Accelerated Vaccine Development and Supply. *Vaccines-Basel* **2021**, *9* (10). DOI: ARTN 1114 10.3390/vaccines9101114.
